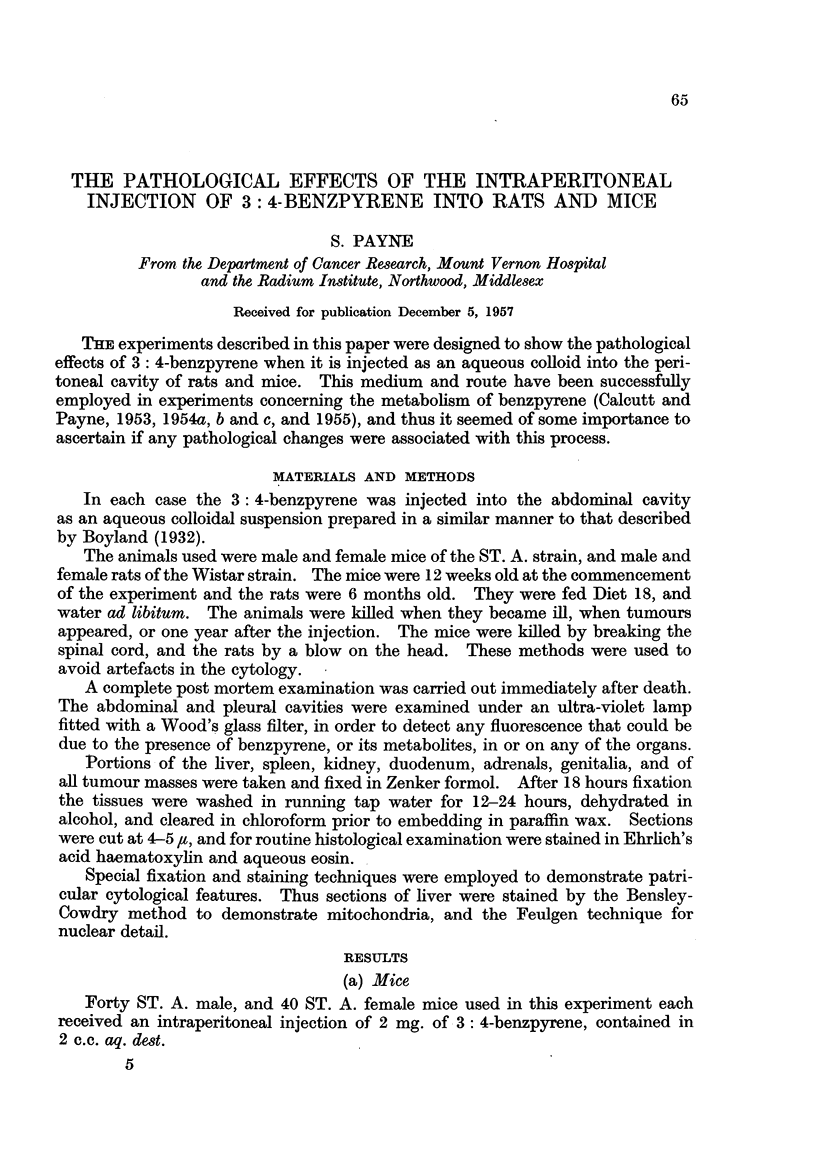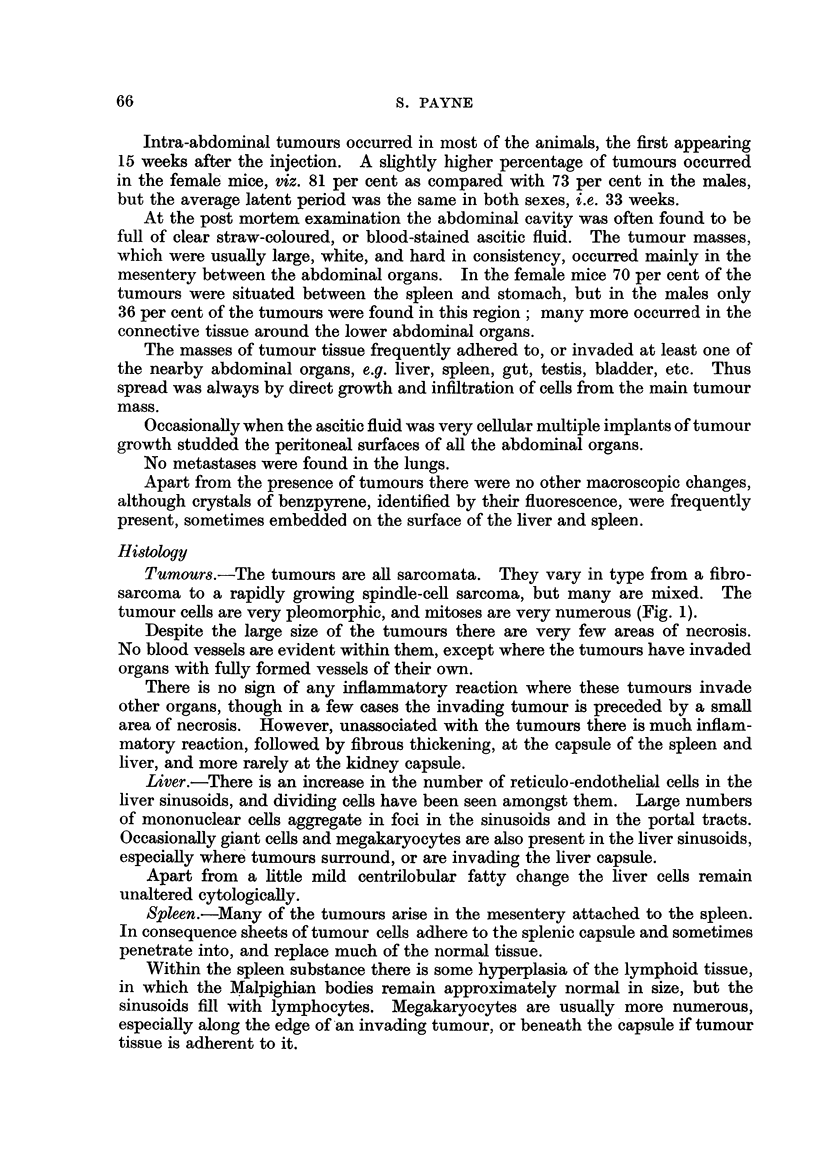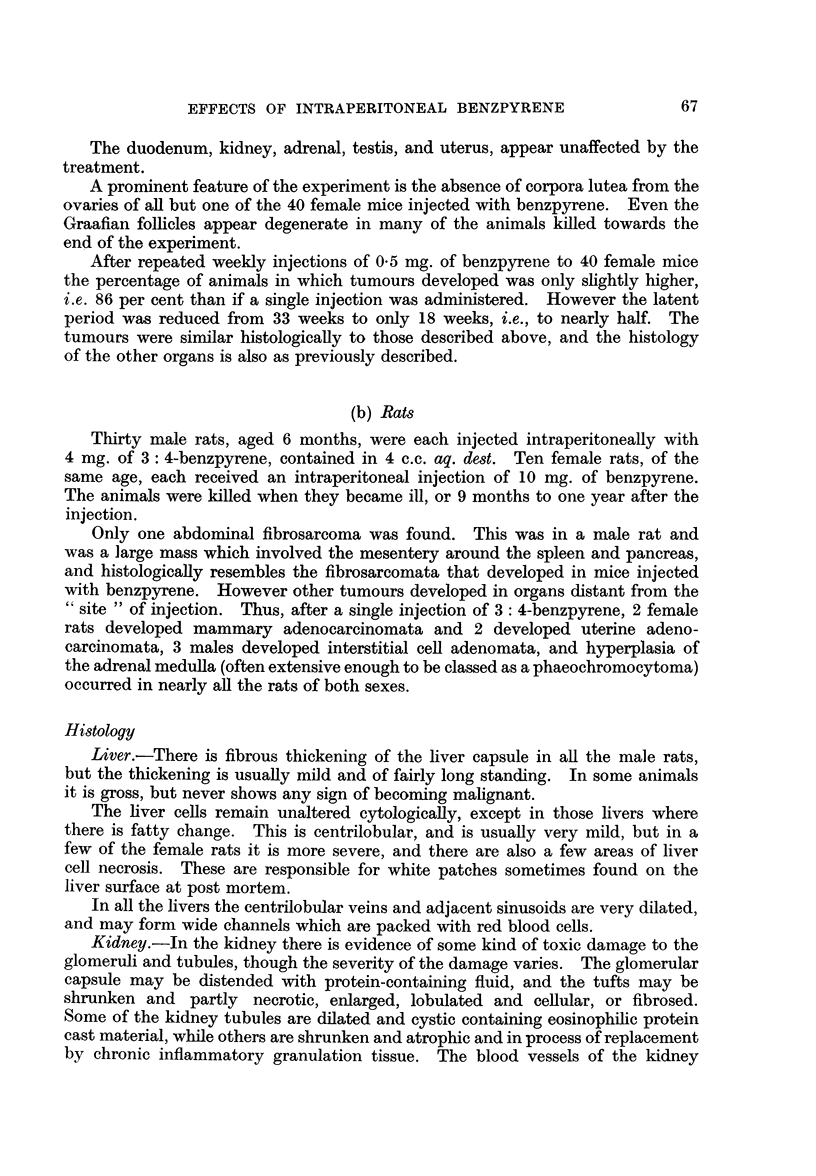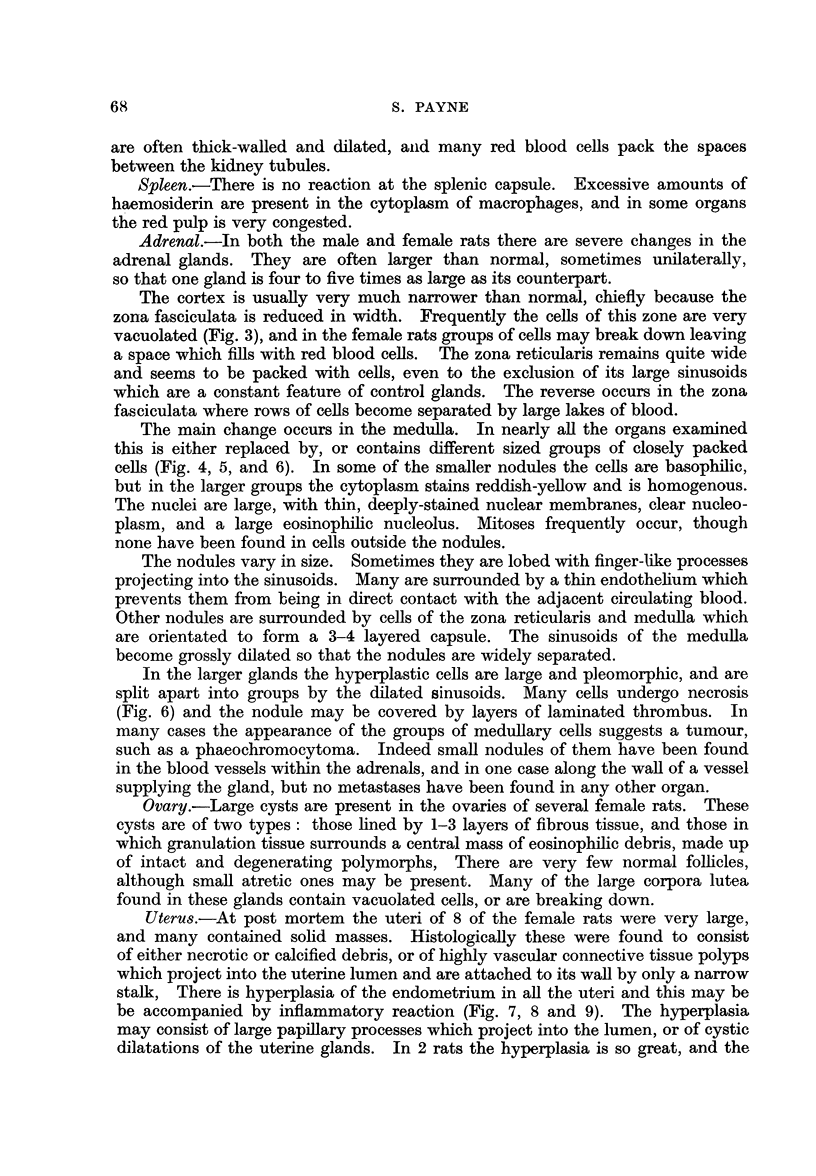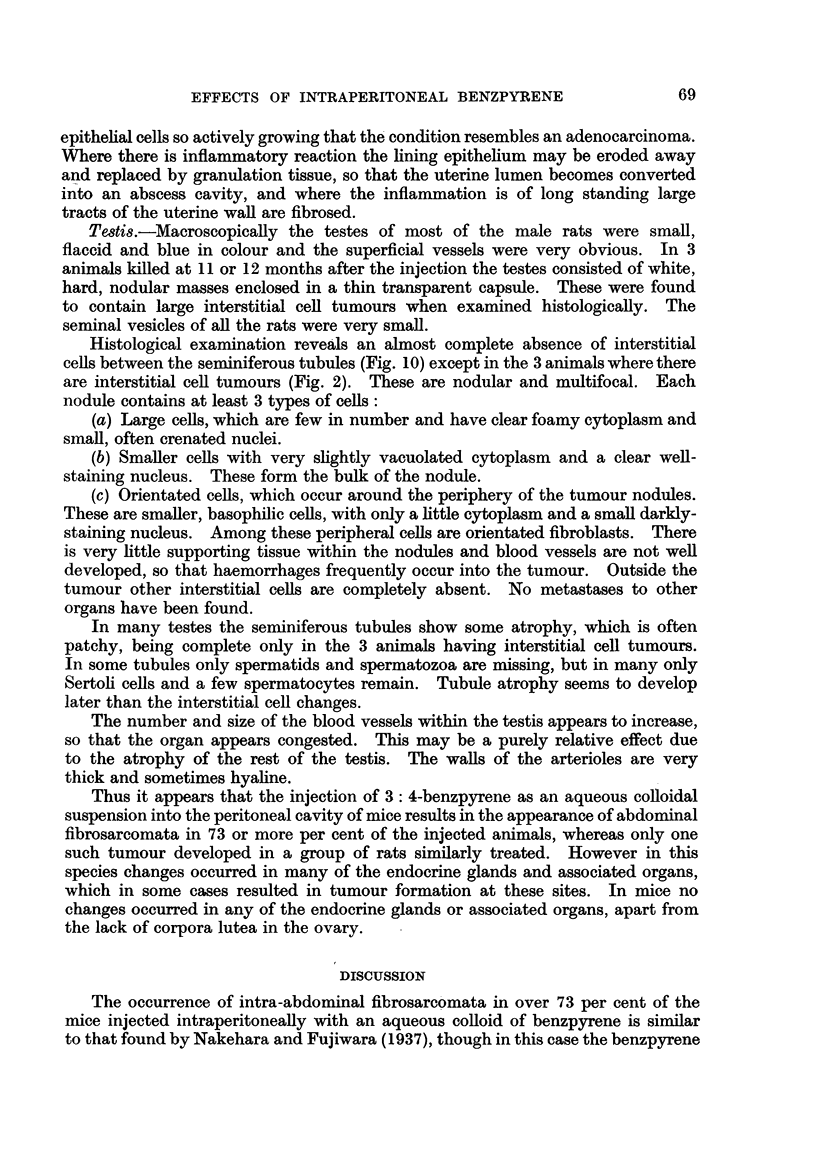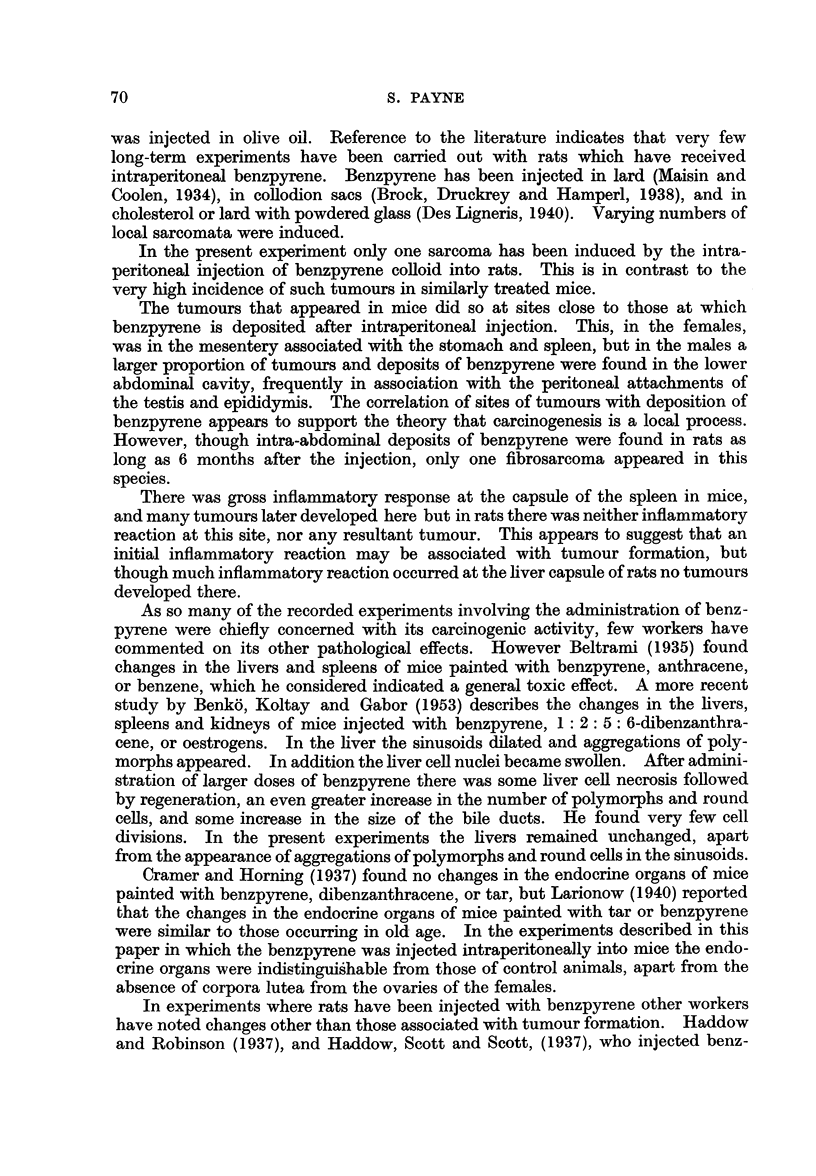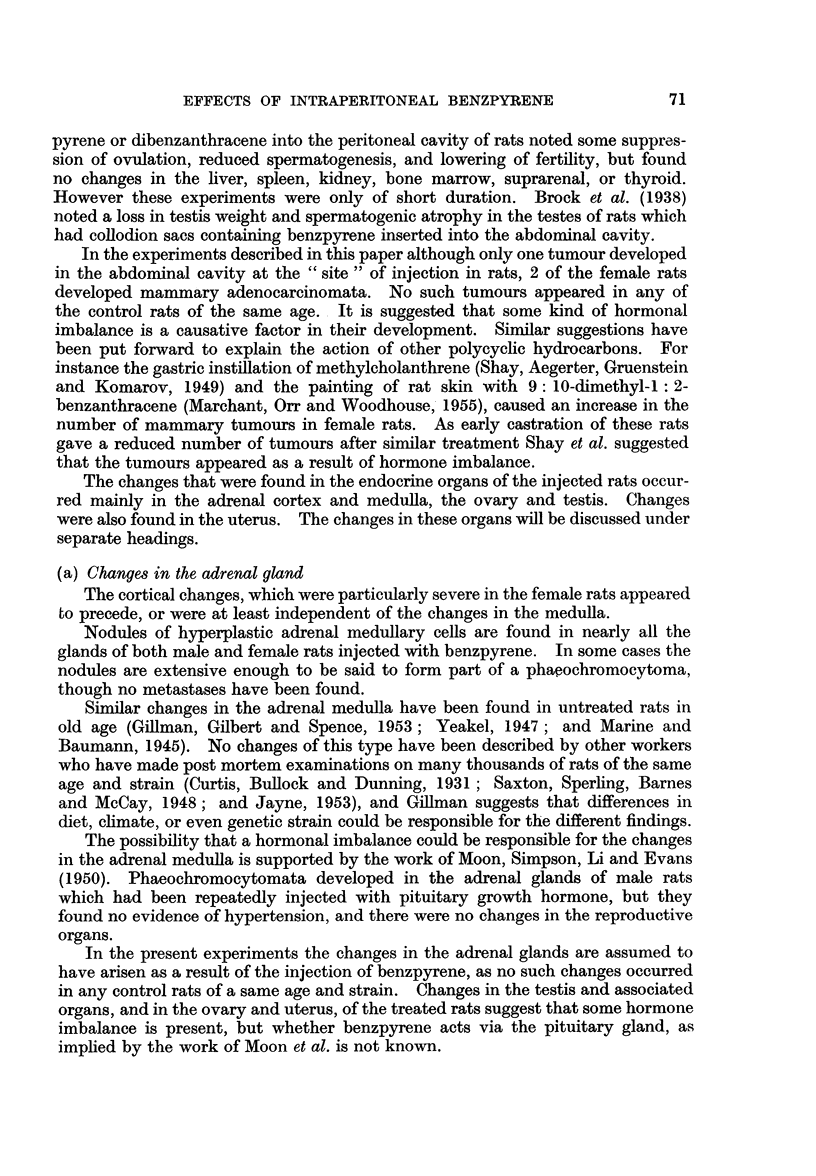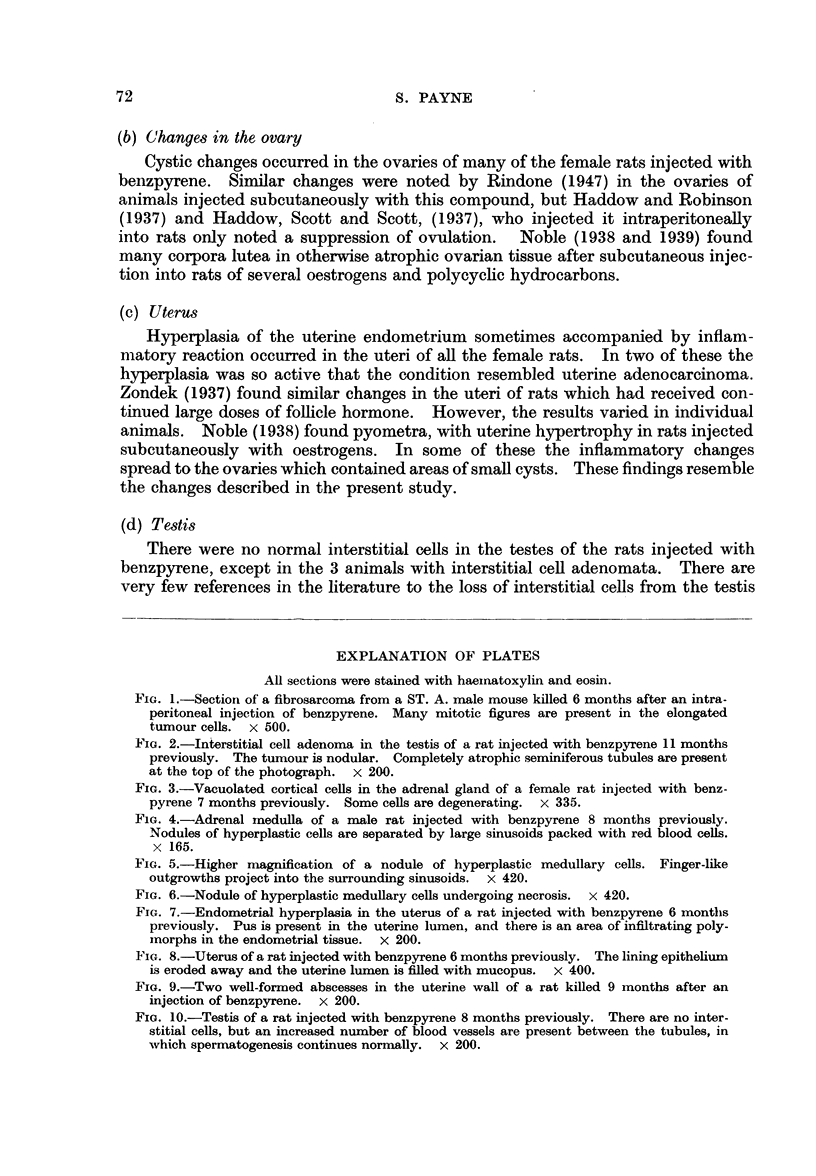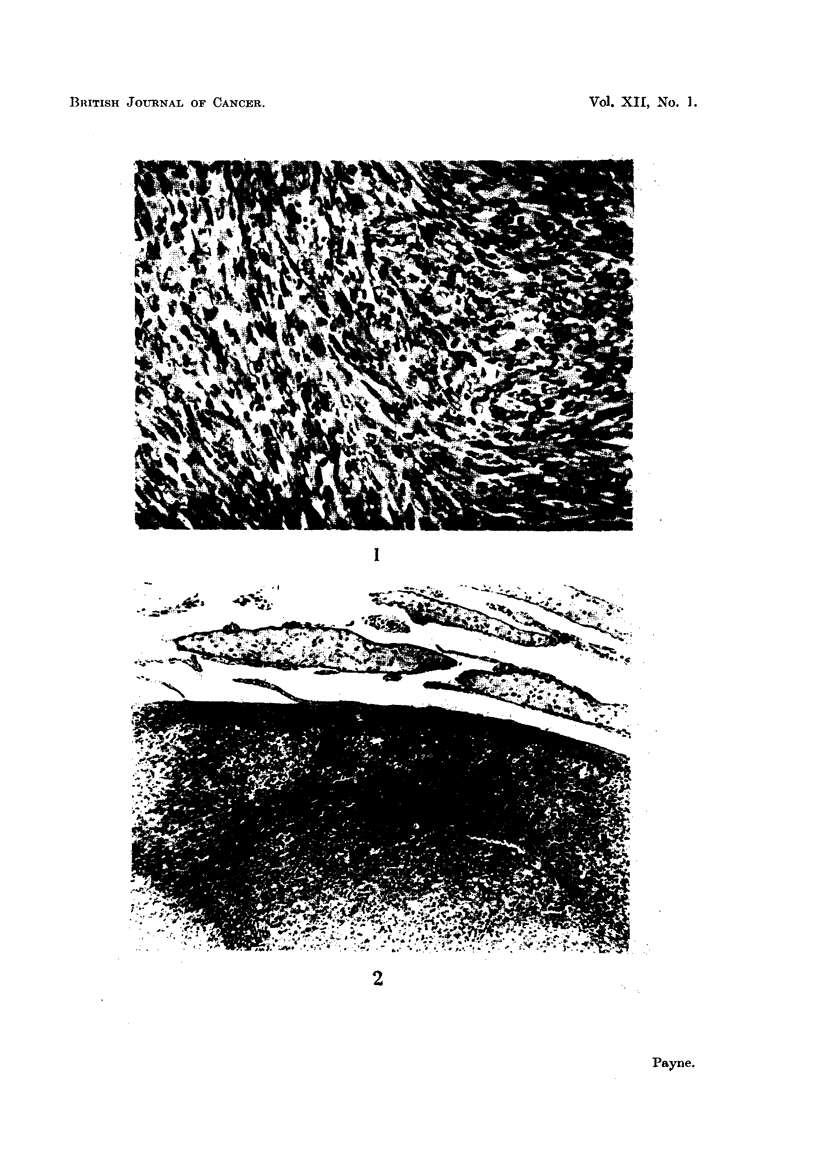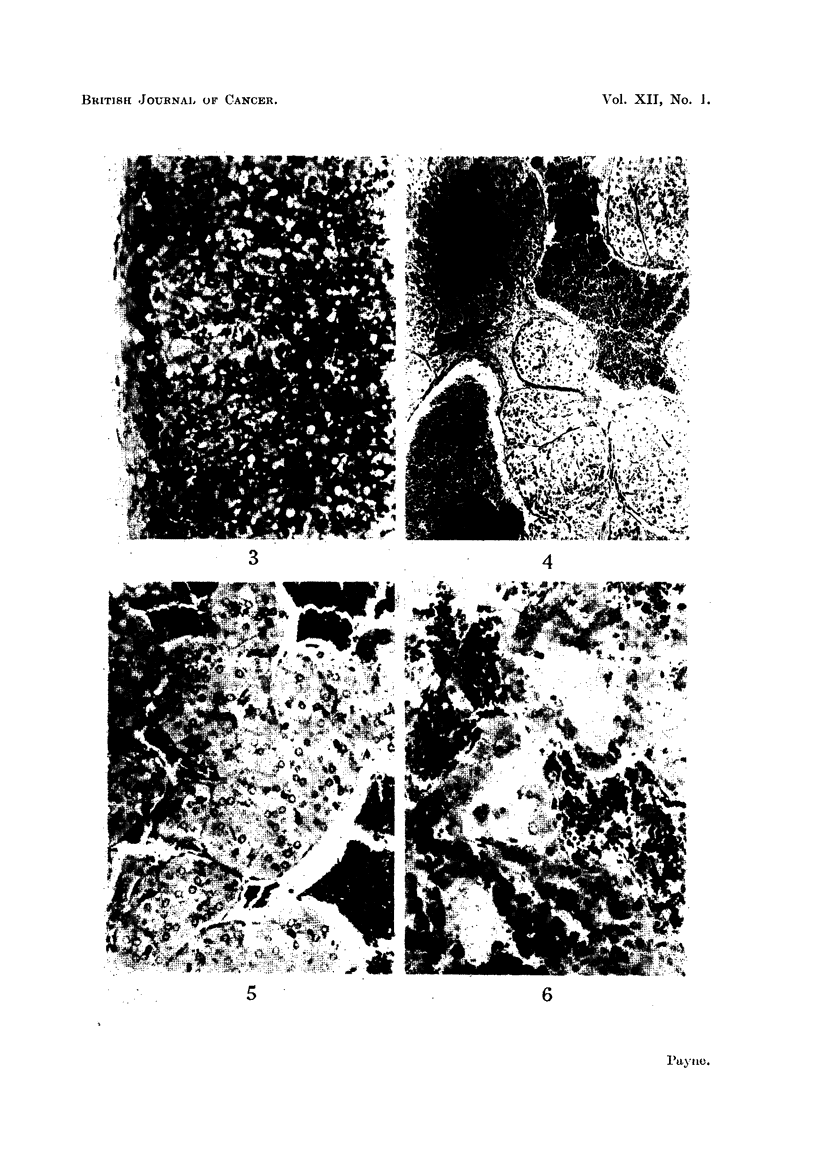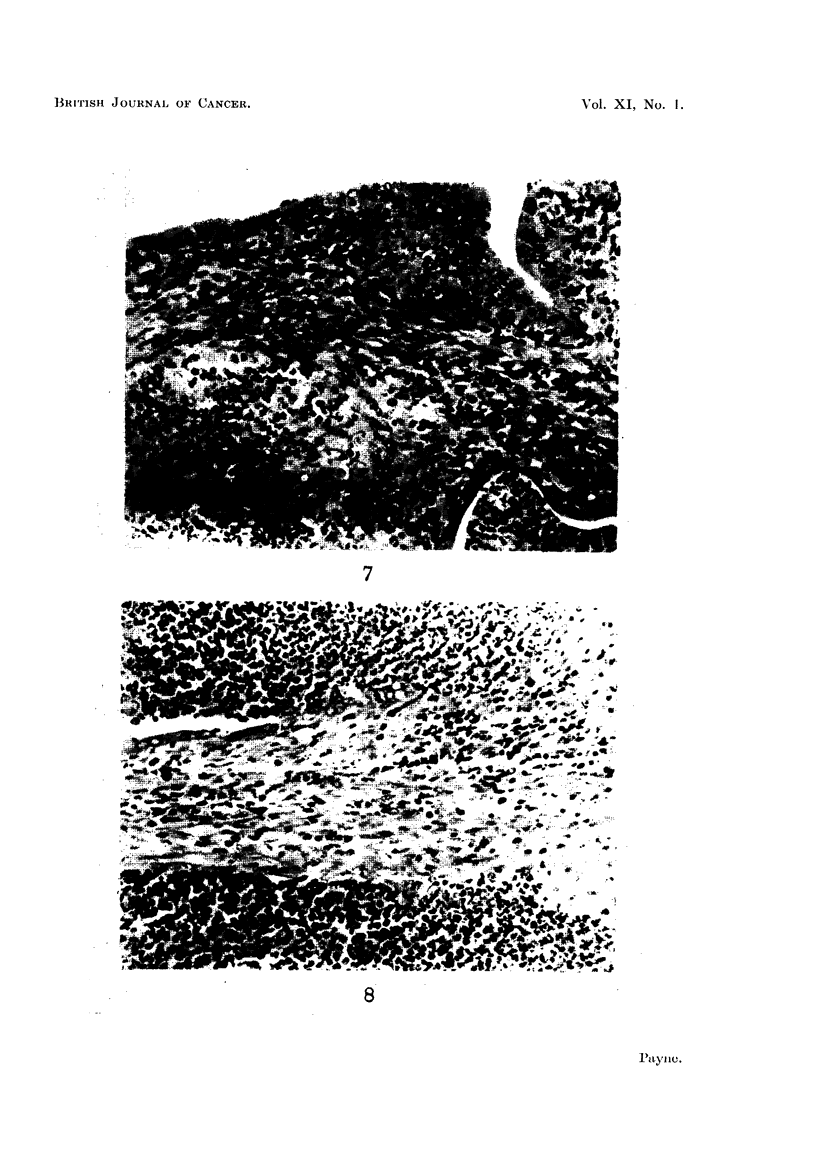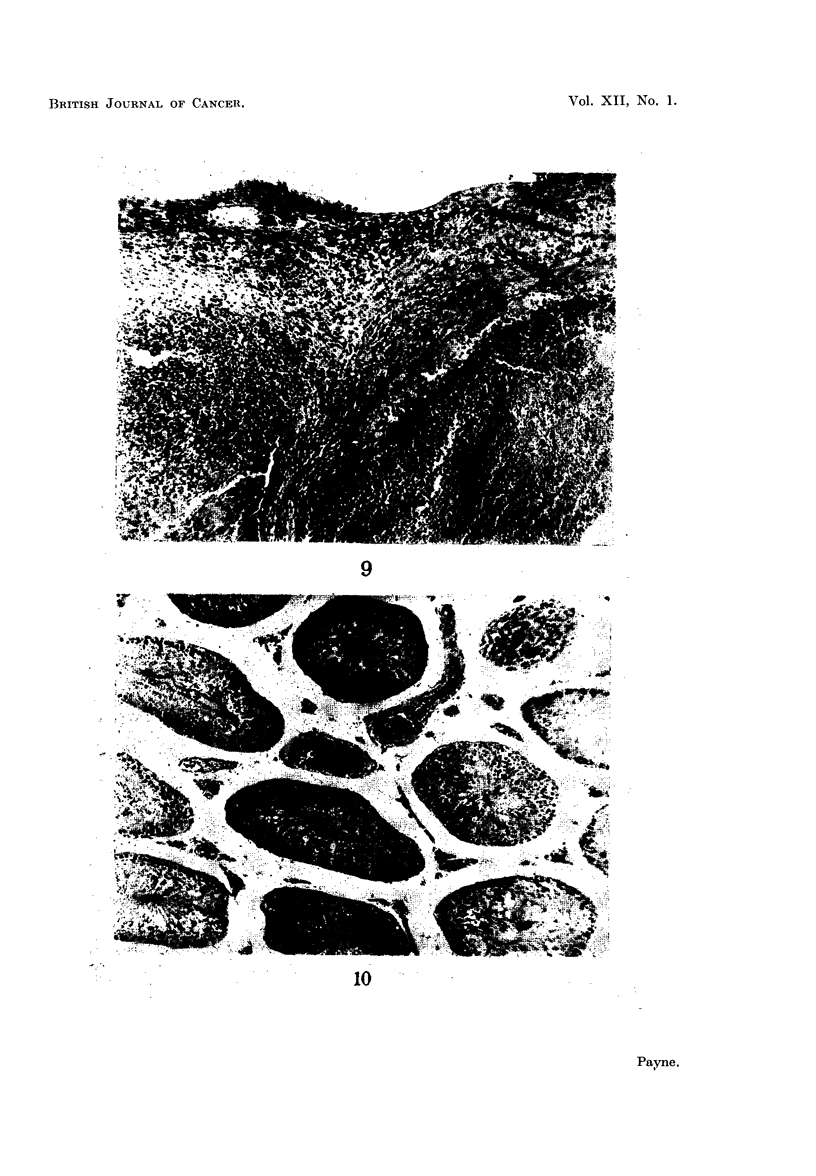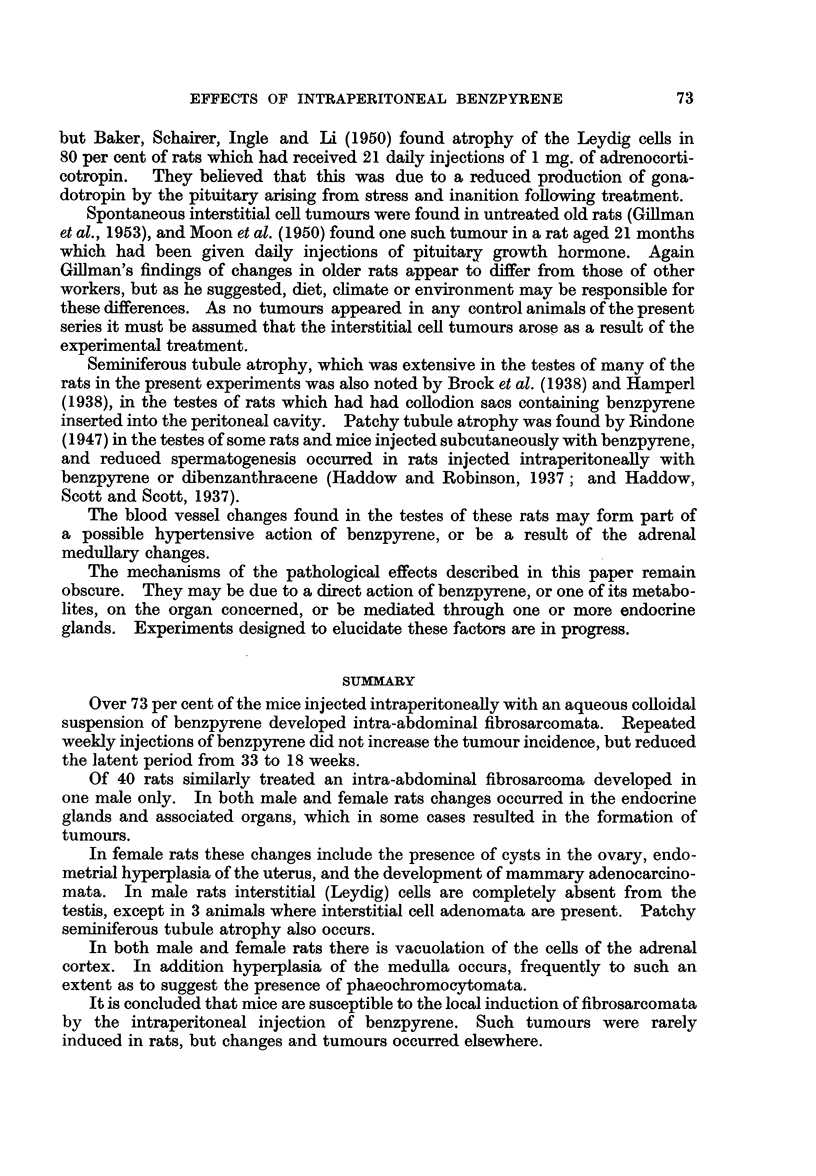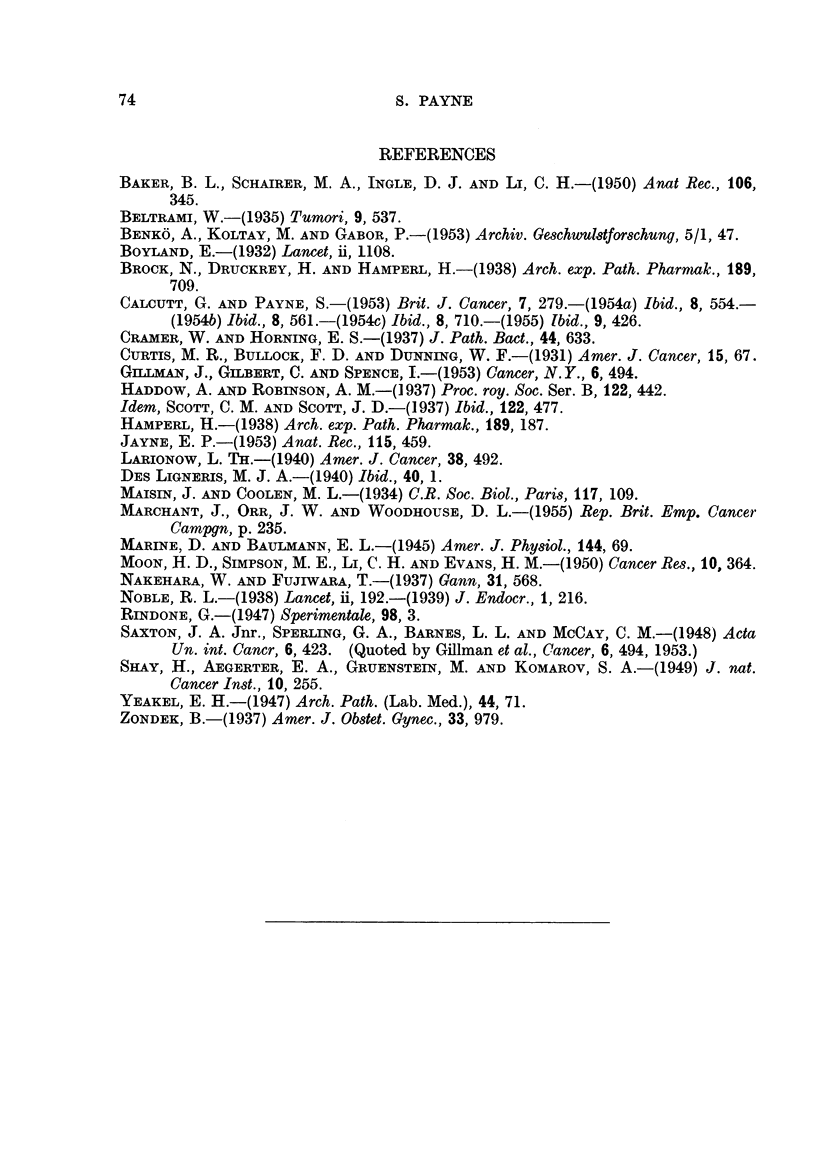# The Pathological Effects of the Intraperitoneal Injection of 3:4-Benzpyrene into Rats and Mice

**DOI:** 10.1038/bjc.1958.10

**Published:** 1958-03

**Authors:** S. Payne

## Abstract

**Images:**


### THE PATHOLOGICAL EFFECTS OF THE INTRAPERITONEAL INJECTION OF 3 : 4-BENZPYRENE INTO RATS AND MICE

S. PAYNE

*From the Department of Cancer Research, Mount Vernon Hospital and the Radium Institute, Northwood, Middlesex*

Received for publication December 5, 1957

THE experiments described in this paper were designed to show the pathological effects of 3 : 4-benzpyrene when it is injected as an aqueous colloid into the peritoneal cavity of rats and mice. This medium and route have been successfully employed in experiments concerning the metabolism of benzpyrene (Calcutt and Payne, 1953, 1954*a*, *b* and *c*, and 1955), and thus it seemed of some importance to ascertain if any pathological changes were associated with this process.

### MATERIALS AND METHODS

In each case the 3 : 4-benzpyrene was injected into the abdominal cavity as an aqueous colloidal suspension prepared in a similar manner to that described by Boyland (1932).

The animals used were male and female mice of the ST. A. strain, and male and female rats of the Wistar strain. The mice were 12 weeks old at the commencement of the experiment and the rats were 6 months old. They were fed Diet 18, and water *ad libitum*. The animals were killed when they became ill, when tumours appeared, or one year after the injection. The mice were killed by breaking the spinal cord, and the rats by a blow on the head. These methods were used to avoid artefacts in the cytology.

A complete post mortem examination was carried out immediately after death. The abdominal and pleural cavities were examined under an ultra-violet lamp fitted with a Wood's glass filter, in order to detect any fluorescence that could be due to the presence of benzpyrene, or its metabolites, in or on any of the organs.

Portions of the liver, spleen, kidney, duodenum, adrenals, genitalia, and of all tumour masses were taken and fixed in Zenker formol. After 18 hours fixation the tissues were washed in running tap water for 12–24 hours, dehydrated in alcohol, and cleared in chloroform prior to embedding in paraffin wax. Sections were cut at 4–5 $\mu$, and for routine histological examination were stained in Ehrlich's acid haematoxylin and aqueous eosin.

Special fixation and staining techniques were employed to demonstrate patricular cytological features. Thus sections of liver were stained by the Bensley-Cowdry method to demonstrate mitochondria, and the Feulgen technique for nuclear detail.

### RESULTS

### (a) *Mice*

Forty ST. A. male, and 40 ST. A. female mice used in this experiment each received an intraperitoneal injection of 2 mg. of 3 : 4-benzpyrene, contained in 2 c.c. *aq. dest.*

Intra-abdominal tumours occurred in most of the animals, the first appearing 15 weeks after the injection. A slightly higher percentage of tumours occurred in the female mice, *viz.* 81 per cent as compared with 73 per cent in the males, but the average latent period was the same in both sexes, *i.e.* 33 weeks.

At the post mortem examination the abdominal cavity was often found to be full of clear straw-coloured, or blood-stained ascitic fluid. The tumour masses, which were usually large, white, and hard in consistency, occurred mainly in the mesentery between the abdominal organs. In the female mice 70 per cent of the tumours were situated between the spleen and stomach, but in the males only 36 per cent of the tumours were found in this region ; many more occurred in the connective tissue around the lower abdominal organs.

The masses of tumour tissue frequently adhered to, or invaded at least one of the nearby abdominal organs, *e.g.* liver, spleen, gut, testis, bladder, etc. Thus spread was always by direct growth and infiltration of cells from the main tumour mass.

Occasionally when the ascitic fluid was very cellular multiple implants of tumour growth studded the peritoneal surfaces of all the abdominal organs.

No metastases were found in the lungs.

Apart from the presence of tumours there were no other macroscopic changes, although crystals of benzpyrene, identified by their fluorescence, were frequently present, sometimes embedded on the surface of the liver and spleen.

*Histology*

*Tumours.*—The tumours are all sarcomata. They vary in type from a fibrosarcoma to a rapidly growing spindle-cell sarcoma, but many are mixed. The tumour cells are very pleomorphic, and mitoses are very numerous (Fig. 1).

Despite the large size of the tumours there are very few areas of necrosis. No blood vessels are evident within them, except where the tumours have invaded organs with fully formed vessels of their own.

There is no sign of any inflammatory reaction where these tumours invade other organs, though in a few cases the invading tumour is preceded by a small area of necrosis. However, unassociated with the tumours there is much inflammatory reaction, followed by fibrous thickening, at the capsule of the spleen and liver, and more rarely at the kidney capsule.

*Liver.*—There is an increase in the number of reticulo-endothelial cells in the liver sinusoids, and dividing cells have been seen amongst them. Large numbers of mononuclear cells aggregate in foci in the sinusoids and in the portal tracts. Occasionally giant cells and megakaryocytes are also present in the liver sinusoids, especially where tumours surround, or are invading the liver capsule.

Apart from a little mild centrilobular fatty change the liver cells remain unaltered cytologically.

*Spleen.*—Many of the tumours arise in the mesentery attached to the spleen. In consequence sheets of tumour cells adhere to the splenic capsule and sometimes penetrate into, and replace much of the normal tissue.

Within the spleen substance there is some hyperplasia of the lymphoid tissue, in which the Malpighian bodies remain approximately normal in size, but the sinusoids fill with lymphocytes. Megakaryocytes are usually more numerous, especially along the edge of an invading tumour, or beneath the capsule if tumour tissue is adherent to it.

The duodenum, kidney, adrenal, testis, and uterus, appear unaffected by the treatment.

A prominent feature of the experiment is the absence of corpora lutea from the ovaries of all but one of the 40 female mice injected with benzpyrene. Even the Graafian follicles appear degenerate in many of the animals killed towards the end of the experiment.

After repeated weekly injections of 0·5 mg. of benzpyrene to 40 female mice the percentage of animals in which tumours developed was only slightly higher, *i.e.* 86 per cent than if a single injection was administered. However the latent period was reduced from 33 weeks to only 18 weeks, *i.e.*, to nearly half. The tumours were similar histologically to those described above, and the histology of the other organs is also as previously described.

### (b) *Rats*

Thirty male rats, aged 6 months, were each injected intraperitoneally with 4 mg. of 3 : 4-benzpyrene, contained in 4 c.c. *aq. dest.* Ten female rats, of the same age, each received an intraperitoneal injection of 10 mg. of benzpyrene. The animals were killed when they became ill, or 9 months to one year after the injection.

Only one abdominal fibrosarcoma was found. This was in a male rat and was a large mass which involved the mesentery around the spleen and pancreas, and histologically resembles the fibrosarcomata that developed in mice injected with benzpyrene. However other tumours developed in organs distant from the " site " of injection. Thus, after a single injection of 3 : 4-benzpyrene, 2 female rats developed mammary adenocarcinomata and 2 developed uterine adenocarcinomata, 3 males developed interstitial cell adenomata, and hyperplasia of the adrenal medulla (often extensive enough to be classed as a phaeochromocytoma) occurred in nearly all the rats of both sexes.

*Histology*

*Liver.*—There is fibrous thickening of the liver capsule in all the male rats, but the thickening is usually mild and of fairly long standing. In some animals it is gross, but never shows any sign of becoming malignant.

The liver cells remain unaltered cytologically, except in those livers where there is fatty change. This is centrilobular, and is usually very mild, but in a few of the female rats it is more severe, and there are also a few areas of liver cell necrosis. These are responsible for white patches sometimes found on the liver surface at post mortem.

In all the livers the centrilobular veins and adjacent sinusoids are very dilated, and may form wide channels which are packed with red blood cells.

*Kidney.*—In the kidney there is evidence of some kind of toxic damage to the glomeruli and tubules, though the severity of the damage varies. The glomerular capsule may be distended with protein-containing fluid, and the tufts may be shrunken and partly necrotic, enlarged, lobulated and cellular, or fibrosed. Some of the kidney tubules are dilated and cystic containing eosinophilic protein cast material, while others are shrunken and atrophic and in process of replacement by chronic inflammatory granulation tissue. The blood vessels of the kidney are often thick-walled and dilated, and many red blood cells pack the spaces between the kidney tubules.

*Spleen.*—There is no reaction at the splenic capsule. Excessive amounts of haemosiderin are present in the cytoplasm of macrophages, and in some organs the red pulp is very congested.

*Adrenal.*—In both the male and female rats there are severe changes in the adrenal glands. They are often larger than normal, sometimes unilaterally, so that one gland is four to five times as large as its counterpart.

The cortex is usually very much narrower than normal, chiefly because the zona fasciculata is reduced in width. Frequently the cells of this zone are very vacuolated (Fig. 3), and in the female rats groups of cells may break down leaving a space which fills with red blood cells. The zona reticularis remains quite wide and seems to be packed with cells, even to the exclusion of its large sinusoids which are a constant feature of control glands. The reverse occurs in the zona fasciculata where rows of cells become separated by large lakes of blood.

The main change occurs in the medulla. In nearly all the organs examined this is either replaced by, or contains different sized groups of closely packed cells (Fig. 4, 5, and 6). In some of the smaller nodules the cells are basophilic, but in the larger groups the cytoplasm stains reddish-yellow and is homogenous. The nuclei are large, with thin, deeply-stained nuclear membranes, clear nucleoplasm, and a large eosinophilic nucleolus. Mitoses frequently occur, though none have been found in cells outside the nodules.

The nodules vary in size. Sometimes they are lobed with finger-like processes projecting into the sinusoids. Many are surrounded by a thin endothelium which prevents them from being in direct contact with the adjacent circulating blood. Other nodules are surrounded by cells of the zona reticularis and medulla which are orientated to form a 3–4 layered capsule. The sinusoids of the medulla become grossly dilated so that the nodules are widely separated.

In the larger glands the hyperplastic cells are large and pleomorphic, and are split apart into groups by the dilated sinusoids. Many cells undergo necrosis (Fig. 6) and the nodule may be covered by layers of laminated thrombus. In many cases the appearance of the groups of medullary cells suggests a tumour, such as a phaeochromocytoma. Indeed small nodules of them have been found in the blood vessels within the adrenals, and in one case along the wall of a vessel supplying the gland, but no metastases have been found in any other organ.

*Ovary.*—Large cysts are present in the ovaries of several female rats. These cysts are of two types : those lined by 1–3 layers of fibrous tissue, and those in which granulation tissue surrounds a central mass of eosinophilic debris, made up of intact and degenerating polymorphs, There are very few normal follicles, although small atretic ones may be present. Many of the large corpora lutea found in these glands contain vacuolated cells, or are breaking down.

*Uterus.*—At post mortem the uteri of 8 of the female rats were very large, and many contained solid masses. Histologically these were found to consist of either necrotic or calcified debris, or of highly vascular connective tissue polyps which project into the uterine lumen and are attached to its wall by only a narrow stalk, There is hyperplasia of the endometrium in all the uteri and this may be be accompanied by inflammatory reaction (Fig. 7, 8 and 9). The hyperplasia may consist of large papillary processes which project into the lumen, or of cystic dilatations of the uterine glands. In 2 rats the hyperplasia is so great, and the epithelial cells so actively growing that the condition resembles an adenocarcinoma. Where there is inflammatory reaction the lining epithelium may be eroded away and replaced by granulation tissue, so that the uterine lumen becomes converted into an abscess cavity, and where the inflammation is of long standing large tracts of the uterine wall are fibrosed.

*Testis.*—Macroscopically the testes of most of the male rats were small, flaccid and blue in colour and the superficial vessels were very obvious. In 3 animals killed at 11 or 12 months after the injection the testes consisted of white, hard, nodular masses enclosed in a thin transparent capsule. These were found to contain large interstitial cell tumours when examined histologically. The seminal vesicles of all the rats were very small.

Histological examination reveals an almost complete absence of interstitial cells between the seminiferous tubules (Fig. 10) except in the 3 animals where there are interstitial cell tumours (Fig. 2). These are nodular and multifocal. Each nodule contains at least 3 types of cells :

(*a*) Large cells, which are few in number and have clear foamy cytoplasm and small, often crenated nuclei.

(*b*) Smaller cells with very slightly vacuolated cytoplasm and a clear well-staining nucleus. These form the bulk of the nodule.

(*c*) Orientated cells, which occur around the periphery of the tumour nodules. These are smaller, basophilic cells, with only a little cytoplasm and a small darkly-staining nucleus. Among these peripheral cells are orientated fibroblasts. There is very little supporting tissue within the nodules and blood vessels are not well developed, so that haemorrhages frequently occur into the tumour. Outside the tumour other interstitial cells are completely absent. No metastases to other organs have been found.

In many testes the seminiferous tubules show some atrophy, which is often patchy, being complete only in the 3 animals having interstitial cell tumours. In some tubules only spermatids and spermatozoa are missing, but in many only Sertoli cells and a few spermatocytes remain. Tubule atrophy seems to develop later than the interstitial cell changes.

The number and size of the blood vessels within the testis appears to increase, so that the organ appears congested. This may be a purely relative effect due to the atrophy of the rest of the testis. The walls of the arterioles are very thick and sometimes hyaline.

Thus it appears that the injection of 3 : 4-benzpyrene as an aqueous colloidal suspension into the peritoneal cavity of mice results in the appearance of abdominal fibrosarcomata in 73 or more per cent of the injected animals, whereas only one such tumour developed in a group of rats similarly treated. However in this species changes occurred in many of the endocrine glands and associated organs, which in some cases resulted in tumour formation at these sites. In mice no changes occurred in any of the endocrine glands or associated organs, apart from the lack of corpora lutea in the ovary.

### DISCUSSION

The occurrence of intra-abdominal fibrosarcomata in over 73 per cent of the mice injected intraperitoneally with an aqueous colloid of benzpyrene is similar to that found by Nakehara and Fujiwara (1937), though in this case the benzpyrene was injected in olive oil. Reference to the literature indicates that very few long-term experiments have been carried out with rats which have received intraperitoneal benzpyrene. Benzpyrene has been injected in lard (Maisin and Coolen, 1934), in collodion sacs (Brock, Druckrey and Hamperl, 1938), and in cholesterol or lard with powdered glass (Des Ligneris, 1940). Varying numbers of local sarcomata were induced.

In the present experiment only one sarcoma has been induced by the intraperitoneal injection of benzpyrene colloid into rats. This is in contrast to the very high incidence of such tumours in similarly treated mice.

The tumours that appeared in mice did so at sites close to those at which benzpyrene is deposited after intraperitoneal injection. This, in the females, was in the mesentery associated with the stomach and spleen, but in the males a larger proportion of tumours and deposits of benzpyrene were found in the lower abdominal cavity, frequently in association with the peritoneal attachments of the testis and epididymis. The correlation of sites of tumours with deposition of benzpyrene appears to support the theory that carcinogenesis is a local process. However, though intra-abdominal deposits of benzpyrene were found in rats as long as 6 months after the injection, only one fibrosarcoma appeared in this species.

There was gross inflammatory response at the capsule of the spleen in mice, and many tumours later developed here but in rats there was neither inflammatory reaction at this site, nor any resultant tumour. This appears to suggest that an initial inflammatory reaction may be associated with tumour formation, but though much inflammatory reaction occurred at the liver capsule of rats no tumours developed there.

As so many of the recorded experiments involving the administration of benzpyrene were chiefly concerned with its carcinogenic activity, few workers have commented on its other pathological effects. However Beltrami (1935) found changes in the livers and spleens of mice painted with benzpyrene, anthracene, or benzene, which he considered indicated a general toxic effect. A more recent study by Benkö, Koltay and Gabor (1953) describes the changes in the livers, spleens and kidneys of mice injected with benzpyrene, 1 : 2 : 5 : 6-dibenzanthracene, or oestrogens. In the liver the sinusoids dilated and aggregations of polymorphs appeared. In addition the liver cell nuclei became swollen. After administration of larger doses of benzpyrene there was some liver cell necrosis followed by regeneration, an even greater increase in the number of polymorphs and round cells, and some increase in the size of the bile ducts. He found very few cell divisions. In the present experiments the livers remained unchanged, apart from the appearance of aggregations of polymorphs and round cells in the sinusoids.

Cramer and Horning (1937) found no changes in the endocrine organs of mice painted with benzpyrene, dibenzanthracene, or tar, but Larionow (1940) reported that the changes in the endocrine organs of mice painted with tar or benzpyrene were similar to those occurring in old age. In the experiments described in this paper in which the benzpyrene was injected intraperitoneally into mice the endocrine organs were indistinguishable from those of control animals, apart from the absence of corpora lutea from the ovaries of the females.

In experiments where rats have been injected with benzpyrene other workers have noted changes other than those associated with tumour formation. Haddow and Robinson (1937), and Haddow, Scott and Scott, (1937), who injected benzpyrene or dibenzanthracene into the peritoneal cavity of rats noted some suppression of ovulation, reduced spermatogenesis, and lowering of fertility, but found no changes in the liver, spleen, kidney, bone marrow, suprarenal, or thyroid. However these experiments were only of short duration. Brock *et al.* (1938) noted a loss in testis weight and spermatogenic atrophy in the testes of rats which had collodion sacs containing benzpyrene inserted into the abdominal cavity.

In the experiments described in this paper although only one tumour developed in the abdominal cavity at the " site " of injection in rats, 2 of the female rats developed mammary adenocarcinomata. No such tumours appeared in any of the control rats of the same age. It is suggested that some kind of hormonal imbalance is a causative factor in their development. Similar suggestions have been put forward to explain the action of other polycyclic hydrocarbons. For instance the gastric instillation of methylcholanthrene (Shay, Aegerter, Gruenstein and Komarov, 1949) and the painting of rat skin with 9 : 10-dimethyl-1 : 2-benzanthracene (Marchant, Orr and Woodhouse, 1955), caused an increase in the number of mammary tumours in female rats. As early castration of these rats gave a reduced number of tumours after similar treatment Shay *et al.* suggested that the tumours appeared as a result of hormone imbalance.

The changes that were found in the endocrine organs of the injected rats occurred mainly in the adrenal cortex and medulla, the ovary and testis. Changes were also found in the uterus. The changes in these organs will be discussed under separate headings.

(a) *Changes in the adrenal gland*

The cortical changes, which were particularly severe in the female rats appeared to precede, or were at least independent of the changes in the medulla.

Nodules of hyperplastic adrenal medullary cells are found in nearly all the glands of both male and female rats injected with benzpyrene. In some cases the nodules are extensive enough to be said to form part of a phaeochromocytoma, though no metastases have been found.

Similar changes in the adrenal medulla have been found in untreated rats in old age (Gillman, Gilbert and Spence, 1953 ; Yeakel, 1947 ; and Marine and Baumann, 1945). No changes of this type have been described by other workers who have made post mortem examinations on many thousands of rats of the same age and strain (Curtis, Bullock and Dunning, 1931 ; Saxton, Sperling, Barnes and McCay, 1948 ; and Jayne, 1953), and Gillman suggests that differences in diet, climate, or even genetic strain could be responsible for the different findings.

The possibility that a hormonal imbalance could be responsible for the changes in the adrenal medulla is supported by the work of Moon, Simpson, Li and Evans (1950). Phaeochromocytomata developed in the adrenal glands of male rats which had been repeatedly injected with pituitary growth hormone, but they found no evidence of hypertension, and there were no changes in the reproductive organs.

In the present experiments the changes in the adrenal glands are assumed to have arisen as a result of the injection of benzpyrene, as no such changes occurred in any control rats of a same age and strain. Changes in the testis and associated organs, and in the ovary and uterus, of the treated rats suggest that some hormone imbalance is present, but whether benzpyrene acts via the pituitary gland, as implied by the work of Moon *et al.* is not known.

(*b*) *Changes in the ovary*

Cystic changes occurred in the ovaries of many of the female rats injected with benzpyrene. Similar changes were noted by Rindone (1947) in the ovaries of animals injected subcutaneously with this compound, but Haddow and Robinson (1937) and Haddow, Scott and Scott, (1937), who injected it intraperitoneally into rats only noted a suppression of ovulation. Noble (1938 and 1939) found many corpora lutea in otherwise atrophic ovarian tissue after subcutaneous injection into rats of several oestrogens and polycyclic hydrocarbons.

(c) *Uterus*

Hyperplasia of the uterine endometrium sometimes accompanied by inflammatory reaction occurred in the uteri of all the female rats. In two of these the hyperplasia was so active that the condition resembled uterine adenocarcinoma. Zondek (1937) found similar changes in the uteri of rats which had received continued large doses of follicle hormone. However, the results varied in individual animals. Noble (1938) found pyometra, with uterine hypertrophy in rats injected subcutaneously with oestrogens. In some of these the inflammatory changes spread to the ovaries which contained areas of small cysts. These findings resemble the changes described in the present study.

(d) *Testis*

There were no normal interstitial cells in the testes of the rats injected with benzpyrene, except in the 3 animals with interstitial cell adenomata. There are very few references in the literature to the loss of interstitial cells from the testis

---

### EXPLANATION OF PLATES

All sections were stained with haematoxylin and eosin.

FIG. 1.—Section of a fibrosarcoma from a ST. A. male mouse killed 6 months after an intraperitoneal injection of benzpyrene. Many mitotic figures are present in the elongated tumour cells. × 500.

FIG. 2.—Interstitial cell adenoma in the testis of a rat injected with benzpyrene 11 months previously. The tumour is nodular. Completely atrophic seminiferous tubules are present at the top of the photograph. × 200.

FIG. 3.—Vacuolated cortical cells in the adrenal gland of a female rat injected with benzpyrene 7 months previously. Some cells are degenerating. × 335.

FIG. 4.—Adrenal medulla of a male rat injected with benzpyrene 8 months previously. Nodules of hyperplastic cells are separated by large sinusoids packed with red blood cells. × 165.

FIG. 5.—Higher magnification of a nodule of hyperplastic medullary cells. Finger-like outgrowths project into the surrounding sinusoids. × 420.

FIG. 6.—Nodule of hyperplastic medullary cells undergoing necrosis. × 420.

FIG. 7.—Endometrial hyperplasia in the uterus of a rat injected with benzpyrene 6 months previously. Pus is present in the uterine lumen, and there is an area of infiltrating polymorphs in the endometrial tissue. × 200.

FIG. 8.—Uterus of a rat injected with benzpyrene 6 months previously. The lining epithelium is eroded away and the uterine lumen is filled with mucopus. × 400.

FIG. 9.—Two well-formed abscesses in the uterine wall of a rat killed 9 months after an injection of benzpyrene. × 200.

FIG. 10.—Testis of a rat injected with benzpyrene 8 months previously. There are no interstitial cells, but an increased number of blood vessels are present between the tubules, in which spermatogenesis continues normally. × 200.

1

2

Payne.

3

4

5

6

7

8

9

10

Payne.

but Baker, Schairer, Ingle and Li (1950) found atrophy of the Leydig cells in 80 per cent of rats which had received 21 daily injections of 1 mg. of adrenocorticotropin. They believed that this was due to a reduced production of gonadotropin by the pituitary arising from stress and inanition following treatment.

Spontaneous interstitial cell tumours were found in untreated old rats (Gillman *et al.*, 1953), and Moon *et al.* (1950) found one such tumour in a rat aged 21 months which had been given daily injections of pituitary growth hormone. Again Gillman's findings of changes in older rats appear to differ from those of other workers, but as he suggested, diet, climate or environment may be responsible for these differences. As no tumours appeared in any control animals of the present series it must be assumed that the interstitial cell tumours arose as a result of the experimental treatment.

Seminiferous tubule atrophy, which was extensive in the testes of many of the rats in the present experiments was also noted by Brock *et al.* (1938) and Hamperl (1938), in the testes of rats which had had collodion sacs containing benzpyrene inserted into the peritoneal cavity. Patchy tubule atrophy was found by Rindone (1947) in the testes of some rats and mice injected subcutaneously with benzpyrene, and reduced spermatogenesis occurred in rats injected intraperitoneally with benzpyrene or dibenzanthracene (Haddow and Robinson, 1937; and Haddow, Scott and Scott, 1937).

The blood vessel changes found in the testes of these rats may form part of a possible hypertensive action of benzpyrene, or be a result of the adrenal medullary changes.

The mechanisms of the pathological effects described in this paper remain obscure. They may be due to a direct action of benzpyrene, or one of its metabolites, on the organ concerned, or be mediated through one or more endocrine glands. Experiments designed to elucidate these factors are in progress.

SUMMARY

Over 73 per cent of the mice injected intraperitoneally with an aqueous colloidal suspension of benzpyrene developed intra-abdominal fibrosarcomata. Repeated weekly injections of benzpyrene did not increase the tumour incidence, but reduced the latent period from 33 to 18 weeks.

Of 40 rats similarly treated an intra-abdominal fibrosarcoma developed in one male only. In both male and female rats changes occurred in the endocrine glands and associated organs, which in some cases resulted in the formation of tumours.

In female rats these changes include the presence of cysts in the ovary, endometrial hyperplasia of the uterus, and the development of mammary adenocarcinomata. In male rats interstitial (Leydig) cells are completely absent from the testis, except in 3 animals where interstitial cell adenomata are present. Patchy seminiferous tubule atrophy also occurs.

In both male and female rats there is vacuolation of the cells of the adrenal cortex. In addition hyperplasia of the medulla occurs, frequently to such an extent as to suggest the presence of phaeochromocytomata.

It is concluded that mice are susceptible to the local induction of fibrosarcomata by the intraperitoneal injection of benzpyrene. Such tumours were rarely induced in rats, but changes and tumours occurred elsewhere.

### REFERENCES

BAKER, B. L., SCHAIRER, M. A., INGLE, D. J. AND LI, C. H.—(1950) *Anat Rec.*, **106**, 345.

BELTRAMI, W.—(1935) *Tumori*, **9**, 537.

BENKÖ, A., KOLTAY, M. AND GABOR, P.—(1953) *Archiv. Geschwulstforschung*, 5/1, 47.

BOYLAND, E.—(1932) *Lancet*, ii, 1108.

BROCK, N., DRUCKREY, H. AND HAMPERL, H.—(1938) *Arch. exp. Path. Pharmak.*, **189**, 709.

CALCUTT, G. AND PAYNE, S.—(1953) *Brit. J. Cancer*, **7**, 279.—(1954*a*) *Ibid.*, **8**, 554.—(1954*b*) *Ibid.*, **8**, 561.—(1954*c*) *Ibid.*, **8**, 710.—(1955) *Ibid.*, **9**, 426.

CRAMER, W. AND HORNING, E. S.—(1937) *J. Path. Bact.*, **44**, 633.

CURTIS, M. R., BULLOCK, F. D. AND DUNNING, W. F.—(1931) *Amer. J. Cancer*, **15**, 67.

GILLMAN, J., GILBERT, C. AND SPENCE, I.—(1953) *Cancer, N.Y.*, **6**, 494.

HADDOW, A. AND ROBINSON, A. M.—(1937) *Proc. roy. Soc.* Ser. B, **122**, 442.

*Idem*, SCOTT, C. M. AND SCOTT, J. D.—(1937) *Ibid.*, **122**, 477.

HAMPERL, H.—(1938) *Arch. exp. Path. Pharmak.*, **189**, 187.

JAYNE, E. P.—(1953) *Anat. Rec.*, **115**, 459.

LARIONOW, L. TH.—(1940) *Amer. J. Cancer*, **38**, 492.

DES LIGNERIS, M. J. A.—(1940) *Ibid.*, **40**, 1.

MAISIN, J. AND COOLEN, M. L.—(1934) *C.R. Soc. Biol., Paris*, **117**, 109.

MARCHANT, J., ORR, J. W. AND WOODHOUSE, D. L.—(1955) *Rep. Brit. Emp. Cancer Campgn*, p. 235.

MARINE, D. AND BAULMANN, E. L.—(1945) *Amer. J. Physiol.*, **144**, 69.

MOON, H. D., SIMPSON, M. E., LI, C. H. AND EVANS, H. M.—(1950) *Cancer Res.*, **10**, 364.

NAKEHARA, W. AND FUJIWARA, T.—(1937) *Gann*, **31**, 568.

NOBLE, R. L.—(1938) *Lancet*, ii, 192.—(1939) *J. Endocr.*, 1, 216.

RINDONE, G.—(1947) *Sperimentale*, **98**, 3.

SAXTON, J. A. Jnr., SPERLING, G. A., BARNES, L. L. AND MCCAY, C. M.—(1948) *Acta Un. int. Cancr*, **6**, 423. (Quoted by Gillman *et al.*, *Cancer*, **6**, 494, 1953.)

SHAY, H., AEGERTER, E. A., GRUENSTEIN, M. AND KOMAROV, S. A.—(1949) *J. nat. Cancer Inst.*, **10**, 255.

YEAKEL, E. H.—(1947) *Arch. Path.* (Lab. Med.), **44**, 71.

ZONDEK, B.—(1937) *Amer. J. Obstet. Gynec.*, **33**, 979.